# Blood-brain barrier and blood-tumoral barrier formation in glioblastoma: endothelial cells and glioblastoma stem cells at the immune-brain crossroad

**DOI:** 10.1186/s12987-026-00850-1

**Published:** 2026-07-27

**Authors:** Maria Rosito, Ruth Lyck, Britta Engelhardt, Cristina Limatola

**Affiliations:** 1 Department of Life Sciences, Health and Health Professions, Link Campus University, 00165 Roma, Italy; 2 Center for Life Nanoscience & Neuroscience, Istituto Italiano di Tecnologia CLN2S, Rome, Italy; 3 Theodor Kocher Institute, University of Bern, Bern, 3012 Switzerland; 4 Department of Physiology and Pharmacology, Laboratory Affiliated to Istituto Pasteur Italia, Sapienza University of Rome, 00185 Rome, Italy; 5 IRCCS Neuromed, 86077 Pozzilli, Italy

**Keywords:** Blood–brain barrier, Blood–tumor barrier, Glioblastoma stem cells, Endothelial cells, Tumor microenvironment, Immune–brain crosstalk, Perivascular niche, Tumor microtubes, Tunneling nanotubes, Vascular remodeling, 3D in vitro models, Microfluidic platforms

## Abstract

Glioblastoma (GBM) is the most aggressive primary brain tumor characterized by significant cellular and molecular complexity. Central to this complexity are glioma stem cells (GSCs) and the perivascular niche, a specialized microenvironment that integrates signals from the systemic circulation with cues arising from tumor development. Acting as a regulatory hub, the perivascular niche influences GSC proliferation, migration, and resistance to therapy through multiple interactions among vascular, immune, and tumor cell populations. In this review, we focus on GSCs and examine the cellular composition and molecular mechanisms operating within the perivascular niche, highlighting key modes of crosstalk that sustain tumor progression. We furthermore critically evaluate experimental strategies used to study this compartment, including 2D and 3D cell cultures, organoids, and in vivo models, discussing their respective strengths and limitations for testing intercellular interactions and therapeutic approaches. A comprehensive understanding of tumor heterogeneity and the interactions between different tumor cell populations, combined with well-characterized experimental models, is essential for deriving mechanistic insights and informing the development of more effective targeted therapies for GBM, ultimately advancing our knowledge of how tumor–microenvironment interactions drive GBM progression.

## Anatomy, origin, and mechanism of vascular malformation

The cerebral vasculature is an extensive and highly intricate network thai is made up of arteries and arterioles, capillaries, venules, and veins. Within the microvascular compartment, it ensures the vital delivery of oxygen and nutrients to the brain parenchyma via the blood–brain barrier (BBB), which is a highly specialized physical and functional interface. Brain microvascular endothelial cells, which constitute the BBB, display specialized properties that limit the free movement of ions, molecules, and cells from the bloodstream into the central nervous system CNS parenchyma, while promoting the clearance of toxic substances from the CNS [1].

The development and maintenance of the BBB characteristics in the CNS rely on the constant crosstalk and interaction with the cellular and acellular elements of the CNS [2, 3]. During development, the unique barrier properties of the brain microvascular endothelial cells are prompted by continuous crosstalk with the neuroectoderm [4–8]. In the adult, the BBB endothelium, pericytes, astrocytes, microglia, neurons, and basal membrane elements are collectively referred to as the neurovascular unit (NVU) [9]. The BBB’s specialized properties are dependent on the correct functioning of the NVU’s individual components but are also influenced by the microenvironment generated by the perivascular cells, such as perivascular fibroblasts, stem cells, and vascular smooth muscle cells (SMCs). These cellular elements, together with the acellular components (endothelial and parenchymal basement membranes), generate the perivascular niche (PVN) [10].

During NVU formation, CNS vascular development is driven by the integration of Wnt/β-catenin signaling and β1-integrin–mediated cell–matrix interactions. Neural progenitors secrete Wnt ligands, particularly Wnt7a and Wnt7b, which activate canonical Wnt signaling in endothelial cells via Frizzled/LRP receptors, leading to β-catenin stabilization and transcription of genes required for CNS-specific angiogenesis and blood–brain barrier differentiation [11, 12]. In parallel, endothelial and perivascular cells interact with the extracellular matrix through β1-integrins, which bind basement membrane components such as laminins and regulate adhesion, migration, and vascular morphogenesis [11].

β1-integrins activate intracellular signaling (e.g., FAK/MAPK) [13] and modulate β-catenin activity, thereby fine-tuning endothelial responses to Wnt signals [12]; while Wnt drives endothelial specialization, integrins ensure its context-dependent regulation during NVU assembly [12–14].

Glioblastomas (GBMs) represent the most aggressive and malignant form of primary brain tumors. After surgical resection, patients treated with conventional therapeutic interventions have a median overall survival of 9–12 months [15, 16]. GBM ontogeny is attributed to a subpopulation of neural stem cells (NSC) or glial precursor cells that undergo activation of oncogenic pathways [17]. The former term “Glioblastoma Multiforme” suggests and underlines the great intra-tumoral heterogeneity that leads to brain invasion in: (1) the white matter tracts, (2) the vasculature (perivascular satellitosis), (3) the leptomeningeal space, and (4) the brain parenchyma [17]. Glial brain tumors are highly angiogenic [18] and their aberrant vasculature strongly characterizes tumor initiation and progression [19]. Therefore, during glioblastoma progression, the perivascular space is remodeled by the presence of cancer cells that locally shape the brain vasculature, activating different strategies of vessel formation.

During development, the formation of new blood vessels may involve the processes of (i) vasculogenesis that relies on the differentiation of a mesodermal angioblast or hemangioblast into an endothelial cell (EC) [20]; (ii) angiogenesis: the formation of a new blood vessel from a pre-existing one [9, 21, 22]; (iii) intussusception: the splitting of an existing blood vessel through the formation of intraluminal tissue pillars [23].

During GBM development, three additional strategies of vasculature remodeling might occur: iv) vascular co-option, where tumor cells grow along pre-existing blood vessels [24–26]; v) GSCs transdifferentiation to ECs or pericytes [27–30], and vi) vascular mimicry, where tumor cells intercalate the blood vessel endothelium, mimicking an EC [22]. Altogether, these mechanisms can also be in continuity with each other in the progression of the primary tumor or during relapses.

In this scenario of GBM progression and vascular remodeling, the BBB is altered and influenced by the presence of GBM cells, which generate a new distinct structure called the blood-tumor barrier (BTB). The tumor cell colonization can thus induce a new microenvironment where the cellular and acellular components are re-educated to support tumor development.

The presence of tumor cells that colonize the NVU generates profound structural changes in this specialized anatomical site, remodeling the extracellular matrix (ECM) and thus providing structural support for migration and activating different pathways that contribute to GBM tolerance and progression [31]. Although the BTB is characterized by a structural rearrangement, effective drug delivery is still challenged by BTB barrier integrity and heterogeneous permeability, which represent the limiting factors of penetration and diffusion of most therapeutic agents [32].

GBM progression and patient survival can be predicted by the molecular signaling and crosstalk between cellular components of the NVU and tumor cells. This prompted us to analyze in more detail the specific strategies used by the glioma stem cells in colonizing and remodeling the perivascular niche, describing the different identified stem cells niches in GBM. In the following sections, we examine how GBM cells exploit and remodel the microvascular compartment—including arterioles, capillaries, and post-capillary venules, with a central focus on brain ECs, extracellular matrix, pericytes, and GSCs at the immune–brain interface. We will discuss mechanisms of intercellular communication via tunneling nanotubes and tumor microtubes across the BTB; immune cell trafficking; and experimental in vitro and in vivo approaches to BTB biology, including iPSC-based systems, 3D platforms, microfluidics, and intravital two-photon imaging.

## The stem cell niche: GSCs evolution and perivascular localization

The prevailing theory on the origin of GBM suggests an evolution from the brain stem endogenous niche located in the subventricular zone (SVZ) [33–36]. Lineage-restricted neural progenitors have been proposed as potential glioblastoma-initiating cells, with gliomagenesis further associated with the progressive accumulation of somatic mutations that contribute to tumor initiation [34, 37].

During tumor progression, the most aggressive GBM cells show a high stemness index that is generally attributed to the presence of GSCs which identify self-replicating pluripotent cell niches that are resistant to therapy and generate tumor recurrence [38]. At least five different stemness niches have been described as responsible for the maintenance of the GSCs population: peri-vascular, peri-arteriolar, peri-hypoxic, peri-immune, and extracellular matrix (ECM) niche [39]. Notably, most of them are associated with the EC that lines the blood vessels.

The establishment of the GSC niche in the peri-arteriolar compartment has been associated to the outer rim of the tunica adventitia [40], which resembles the hypoxic conditions [41].

**Fig. 1:**
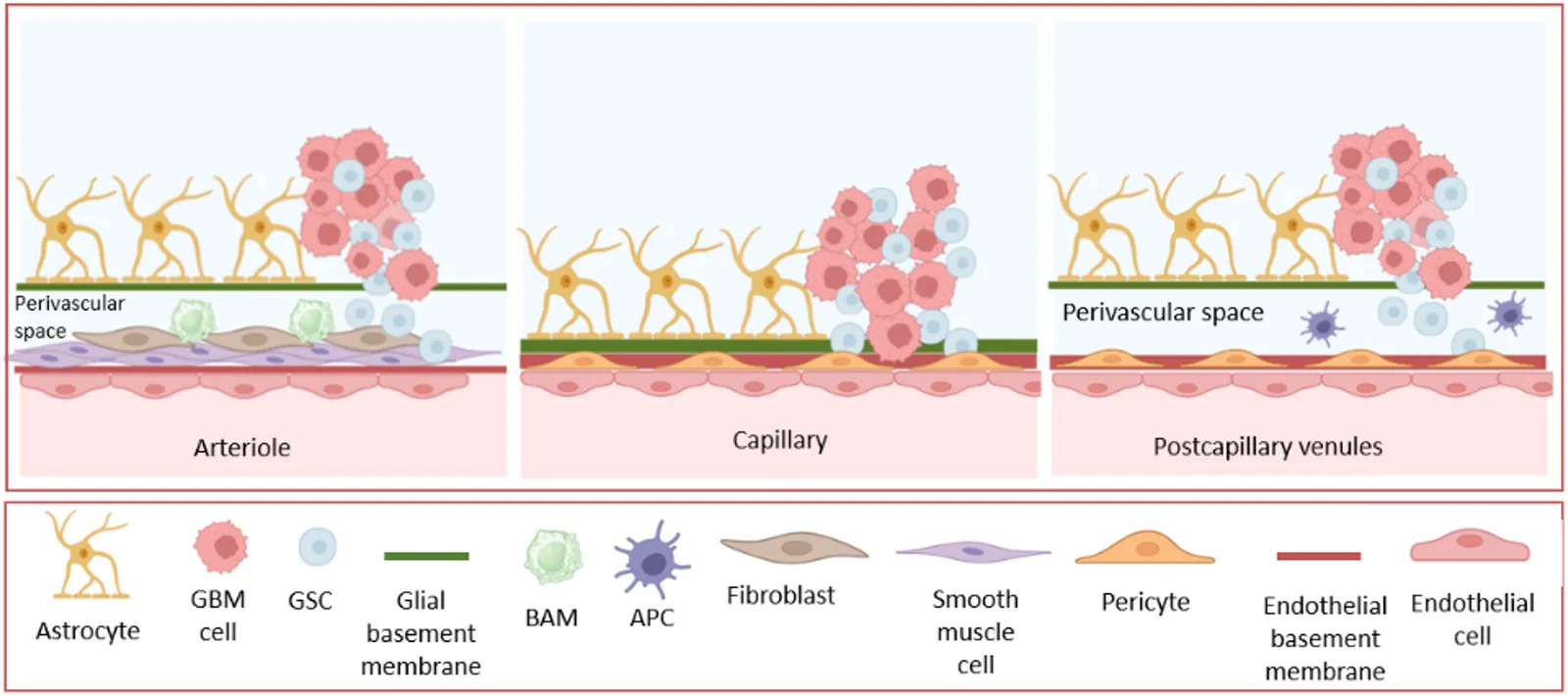
Segment-Specific Differences in Cellular Composition and Spatial Organization of Arterioles, Capillaries, and Post-Capillary Venules During Brain Tumor Progression. Beyond their direct interactions with parenchymal cells, GBM cells and GSCs engage with nearly all cellular and acellular components of specific segments of the vascular tree, including arterioles, capillaries, and postcapillary venules. At arterioles and postcapillary venules, GBM cells and GSCs can interact with border-associated macrophages (BAMs) and antigen-presenting cells (APCs), respectively, before encountering smooth muscle cells (SMCs) and fibroblasts, which gradually transition into a pericyte layer embedded within the endothelial basement membrane at the capillary and postcapillary levels. A precise characterization of GSC localization during tumor progression would be crucial for defining the segment-specific phenotypes they acquire and could guide the development of more effective therapeutic strategies to overcome chemoresistance. Created with BioRender.com

Due to the difficulty of studying the exact location and distribution of cancer cells during the evolution and progression of the disease, we still need to deepen our knowledge of the role and behavior of GSCs in the perivascular niche and the cross-talk that occurs among all the different cell types that constitute the arteriolar, capillary and post-capillary structures (Fig. 1).

In several studies, the GSCs associated with the hypoxia niche have been correlated to high chemoresistance, able to promote the stemness-like phenotype as revealed by high expression of CD133 marker after GSCs irradiation [42, 43].

Another important characteristic of GSCs is the invasive and migratory phenotype [44] that has been linked to hyaluronic acid expression and ECM remodeling [45]. Since the behavior of the stem cell niches is guided by many soluble signaling molecules and ECM remodeling, in the next section, we will discuss these aspects in more detail.

### GSCs and brain ECs crosstalk

The complex architecture of the PVN has been proposed as a GSCs-ECs interaction hub. Through autocrine and paracrine factors, this niche supports GSCs survival and self-renewal properties of the GSCs populations [31, 46]. The PVN thus generates the optimal microenvironment where the GSCs can sense the soluble factors released by ECs and, thanks to the ECM, can anchor, migrate, and establish a cross-talk with the ECs. This intercellular communication not only guarantees the GSCs cell survival but is also important for determining the GSCs differentiation.

In this complex microenvironment, in addition to the GSCs characterization, there is an unmet need to better understand the molecular alteration that occurs in microvascular ECs in and close to the tumor that differentially express different sets of influx and efflux transporters, thus limiting the drug access to the brain [47]. Although we will not go over the drug delivery topic in this section, we want to discuss the interaction between GSCs and brain microvascular endothelial cells during cancer development.

To this end, to characterize potential alterations of the brain microvasculature associated with a GBM lesion [32], Xie and colleagues described key molecular features of CD31 + cells isolated from human GBM surgical resections, identifying different brain ECs clusters addressed to the GBM core and peritumoral tissue. Using single-cell RNA sequencing, the authors clustered five distinct CD31 + ECs phenotypes isolated from GBM resection and peripheral brain tissue with normal histology, leading to the conclusion that some of these clusters exhibit a normal or increased expression of junctional molecules that resemble an intact BBB phenotype [48]. However, other studies reported that in brain ECs ABCB1 (P-glycoprotein) expression is downregulated in the vasculature of GBM [49, 50]. Conversely, several BBB-associated drug transporters [51]—including SLC22A3, SLC36A1, SLC28A1, SLC29A4, and SLC43A3—are upregulated in microvascular ECs isolated from the peritumoral region compared with those in a physiological BBB context.

Taken together, these findings provide only a partial representation of the cellular phenotypes present within these surgical specimens and highlight the current limitation in resolving EC phenotypes across distinct zones of the brain vasculature with sufficient detail. This highlights the marked disorganization of the tumor region driven by GBM progression and indicates that high-resolution spatial transcriptomic approaches will be essential to accurately link cellular diversity with the phenotypic and spatial organization of the tumor microenvironment [52].

At the level of cellular communication and signaling, several in vitro studies have been conducted to investigate the mechanisms through which GBM-derived signals influence this interaction by controlling the secretion of signaling mediators, supporting the idea that GBM cells actively regulate EC activity. These studies often use endothelial-like cell lines to explore the complex microenvironment as a key regulator of GSC–EC crosstalk.

An intriguing mechanism of communication between glioma GSCs and ECs has been described in vitro, showing that POSTN—enriched in extracellular vesicles released by human microvascular endothelial cells (HMECs) following irradiation—may act as a key mediator of GSC migration and their transdifferentiation into endothelial-like cells [53]. This mechanism is added to other roles previously described, in which POSTN promotes GSC niche establishment and the development of a pro-tumoral [54] and pro-angiogenic microenvironment [55].

Among the brain tumor molecules able to shape the microenvironment, Filamin-B, enriched in the EVs released by GSCs, induces HMECs reorganization and migration [53]. In addition, histamine has been identified as the metabolite that is released in large quantities by GSCs and, acting on HBMECs, promotes tumor vasculogenesis [56]. Another mechanism involves the role of TGF-β release by GSCs and its effect on TGF-β receptor III (TβRIII), which is expressed by the glioma cell lines and human brain-derived microvascular endothelial cells (hCMECs).

The interaction of this receptor with the TGF-β superfamily members is associated with decreased survival of LN-229 tumor-bearing mice [57]. The LN-229 cell line is generally considered to exhibit characteristics of predominantly differentiated GBM cells. However, LN-229 cells express transforming growth factor-β receptor III (TβRIII), making them a suitable model for investigating TGF-β-mediated signaling pathways in promoting tumor vascularization. In this context, the reported findings provide insight into mechanisms underlying the phenotypic plasticity and transdifferentiation of GBM cells, processes that may contribute to the acquisition of vascular-like properties. However, to better describe the effect of the TGF-β signaling on the process of tumor vascularization, it will be important to consider the correct luminal or abluminal expression of the receptors on brain ECs and the contribution of the soluble form of the TβRIII that is released by the GBM cells to promote TGF-β2/Smad2 tumorigenic signaling.

Most studies examining GSC–EC crosstalk describe mechanisms based on interactions between patient-derived GSCs and generic HMECs or human cerebral microvascular endothelial cells (hCMECs). In this context, cell-based in vitro models aimed at better dissecting intercellular communication are essential to elucidate the cellular mechanisms underlying this crosstalk.

### GSCs and ECM remodeling

The complex GBM microenvironment is also characterized by an increased ECM deposition and remodeling that occurs during tumor progression. A significant population of GSCs can generate mesenchymal stem cells [30] that, after induction by GBM-secreted CD40L, promote ECM remodeling [58]. GBM samples collected from surgical resection, exhibit specific ECM protein signatures characterized by high proteoglycan and glycoprotein diversity which contributes to the increased tissue stiffness [59]. A recent study described a mechanism of ECM remodeling in GBM driven by the RUNX1/NPM1/H3K4me3 complex, which promotes the transcriptional activation of ECM-associated genes, including FN1, COL4A1, and LUM, coding for Fibronectin 1, Collagen type IV alpha 1 chain, and Lumican, respectively [60].

The interaction between GBM cells and the ECM, together with the activation of mechanotransduction pathways, leading to changes in cell shape, migration, and differentiation, is complex and remains incompletely understood. The cellular focal adhesion expression is actively influenced by matrix stiffness, leading to cytoskeleton remodeling [61]. The importance of ECM remodeling in GSCs adaptation to matrix stiffness has been recently described: the protein kinase R (PKR)-like endoplasmic reticulum kinase (PERK) is actively involved in the detection of stiffness changes via focal adhesion complex formation and plays an important role in the stemness niche maintenance and differentiation process [62, 63].

All these insights into ECM composition and remodeling highlight the importance of physiologically relevant in vitro models for studying GBM biology and crosstalk.

### GSCs and pericytes

Pericytes are important elements of the NVU, are embedded in the endothelial BM of CNS capillaries and venules, and regulate many physiological vascular functions [64]. Pericytes are important for blood vessel formation, maintenance of the BBB, regulation of immune cell entry into the CNS, and they are actively involved in the control of blood flow [65–67].

At the capillary level, pericytes actively contribute to the basement membrane formation and control the ECs cell cycle [68]. In the adult CNS, these cells are able to activate angiogenic programs by secreting VEGF-A and neurogenic locus notch homolog protein (NOTCH) 3 [69].

In GBM, pericytes are actively involved in tumor progression, specifically in ECM remodeling [70] and in releasing chemokines that interfere with the DNA damage repair system [71] and GSCs recruitment [72].

High pericyte density, in surgical resections from GBM patients, has been associated with poorer prognosis [54], likely contributing to vascular stabilization and tumor resistance. The high pericyte number may derive from GSCs recruited through the CXCL12/CXCR4 axis and capable of differentiating into pericytes [28]. In this context, targeting pericytes emerged as a potential therapeutic strategy. Notably, a CD19 CAR-engineered iNK cells approach aimed at pericyte depletion has recently been proposed in one GBM spheroid model [72].

The unique pericyte localization and function suggest that greater attention should be paid to the role of these cells in the GBM microenvironment. Surgical resection from GBM patients revealed the presence of pericytes characterized by high expression of fibroblast activation protein (FAP) responsible for a complex ECM rich in collagen I and fibronectin. This is of interest, also considering that ECM composition is crucial for facilitating the dissemination of glioma cells through the focal adhesion kinase signaling [70].

## Tunnel nanotubes and tumor microtubes: communication bridges across the BTB

Tumor microtubes (TMs) and Tunneling nanotubes (TNT) represent important routes of intercellular communication over long distances, allowing the exchange of organelles, ions, vesicles, and pathogens between nonadjacent cells [27, 73–78]. In GBM, multiple studies have revealed emerging roles for these structures in tumor development and progression, in addition to the contributions of soluble factors, microvesicles, and exosomes released by GBM and host cells.

The first evidence regarding the expression and function of these thin membrane tubes in brain tumors in vivo was provided by Osswald and colleagues, in the maintenance of calcium homeostasis, assuming that TMs are able to distribute calcium ions in large tumor-connected cells, thus avoiding critical and pro-apoptotic calcium level elevation [79]. More recently, it has been proposed that calcium transients in GBM cells control TM dynamics and invasion speed [80] and that TMs-connected cells have been associated with a stemness feature [48]. To underline the association between TM-positive cells and their stemness features together with the recurrence of GBM, Xie and colleagues showed that only nestin-positive cells can extend more TMs after radiotherapy, thus highlighting the association between TMs, stemness, and tumor resistance mechanisms [48].

The functionality of TMs is mediated by connexin 43, a gap junction protein crucial for cell interconnection that appears to confer resistance to therapies, a resistance that is lost upon inhibition of TM-inducers such as Cx43, GAP43, and TTYH [79, 81–83].

GSCs isolated from surgical resection also generate TNT, thin continuous protrusion membranes made by actin, and are negative for tubulin staining, which are functional for mitochondria transfer in GBM-derived tumor organoids [84]. In this model, the co-existence of TMs and TNT is associated with a cooperative networking strategy orchestrated by GBM/GSCs [84]. Another feature of GSCs is the activation of the TGFβ1 pathway that transcriptionally regulates the ECM protein Trombospondin 1 expression: an important mediator of MTs formation [85, 86].

Although the association between GSCs and TNT/TMs formation has been partially clarified, the role of the specific temporal and spatial input coming from the host cells has not yet been investigated. There is currently no proof that TNTs are formed between the brain ECs. Moreover, only scarce evidence exists about the role of TNTs in mediating communication between ECs and other components of the BBB, such as pericytes and astrocytes [87].

**Fig. 2:**
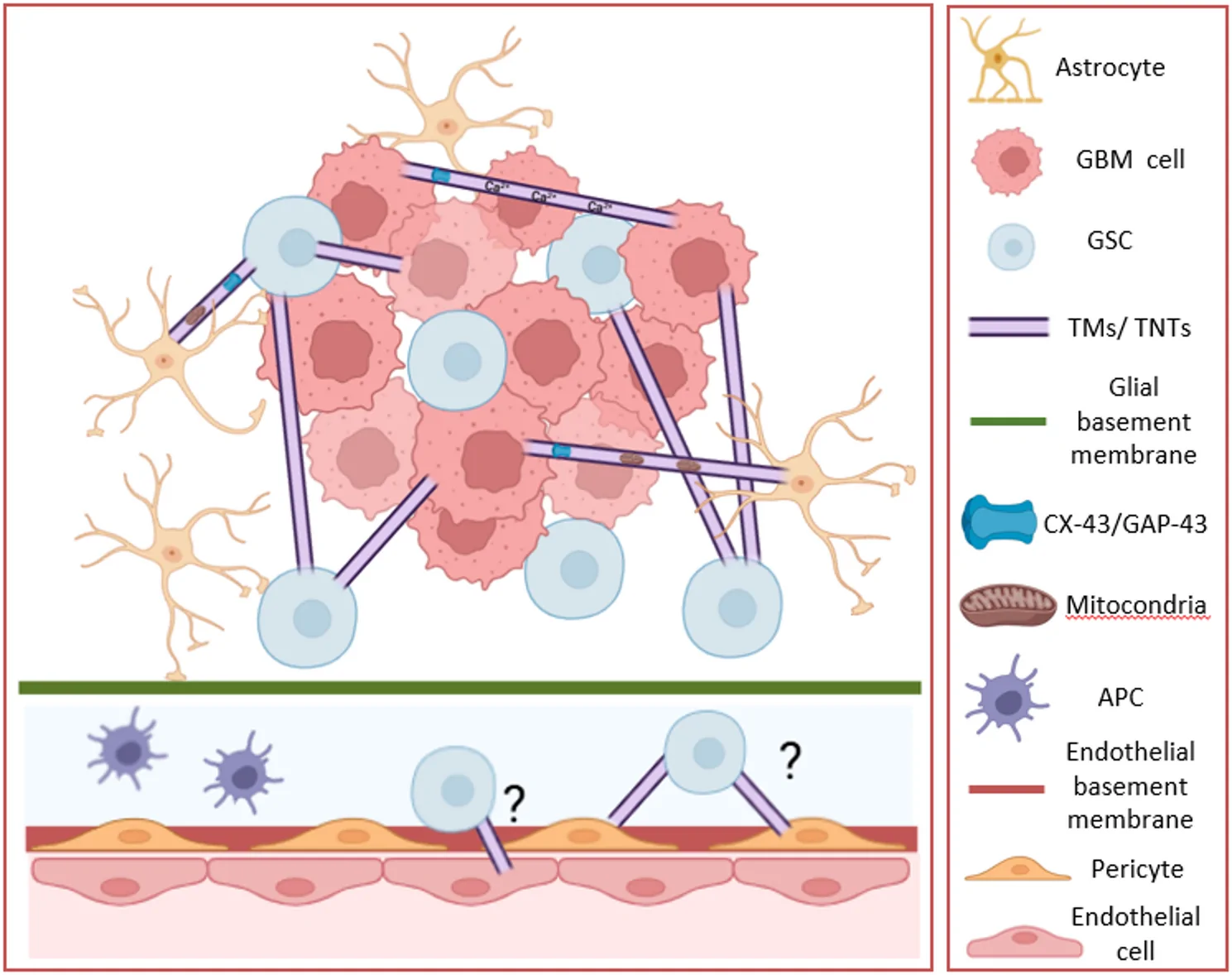
TMs and TNTs in GBM: Intercellular Communication and Potential Links to Neurovascular Unit Components. TMs and TNTs connect the various cellular components of the brain parenchyma and the neurovascular unit. In particular, GSCs seem to play a more prominent role in TM formation. Through connexin 43 (Cx43)-based gap junctions and growth-associated protein 43 (GAP43), these structures enable the exchange of calcium (Ca2^+^) ions and mitochondria between astrocytes and GBM cells. However, it remains unclear whether similar protrusions are also formed to mediate communication between GSCs and endothelial cells or pericytes. Created with BioRender.com

Given the marked heterogeneity of the GBM cell population, greater attention should be devoted to clarifying the stemness status of GSCs in relation to the protrusions that connect GBM cells with other brain parenchymal cells, or other cells belonging to the neurovascular unit. From the perspective of achieving a deeper understanding of how the perivascular stem cell niche of GSCs is established, future studies should more thoroughly investigate the interactions occurring among these cellular populations and determine the specific vascular segments within the vascular tree where these interactions take place (Fig. 2).

## Immune cell trafficking across the BTB

The CNS is an immune-privileged organ where the brain barriers limit immune cell migration and CNS immune surveillance to CNS border zones like the subarachnoid and perivascular spaces [88, 89]. In GBM, non-neoplastic cells such as tumor-associated macrophages (TAMs), T cells, and dendritic cells infiltrate the tumor mass, but their antitumor immune response is strongly impaired due to both intrinsic and extrinsic signals from the tumor microenvironment [90–92]. In this scenario, the cross-talk between the tumor, brain resident cells, and infiltrating immune cells can determine immune suppression, thus influencing tumor development [93, 94].

**Fig. 3:**
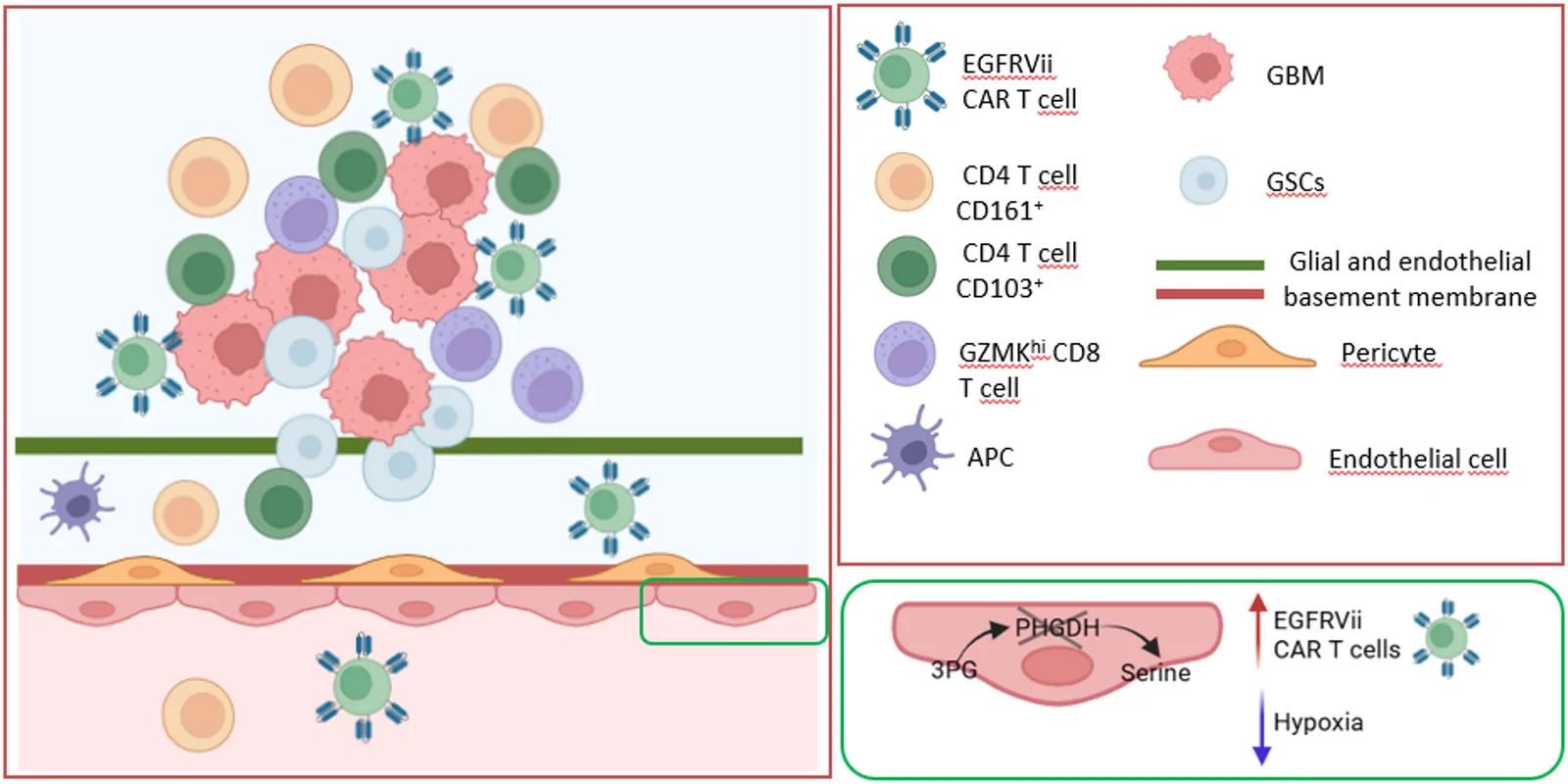
T-cell infiltration and immunometabolic modulation across the BTB. During GBM development, the immune microenvironment encompasses a wide spectrum of T-cell states. Among the CD4 and CD8 T cells, in this figure, we highlight specific CD4 and CD8 T-cell subsets enriched within the tumor mass, such as CD4 CD103 + and CD161 + T cells, which exert an immunosuppressive activity within the TME. GZMKhi CD8 effector T cells are found localized near the vasculature. A promising approach to enhance T cell–based immunotherapy is to disrupt PHGDH-mediated EC metabolism (illustrated in the green box). Inhibition of PHGDH has been shown to reduce tumor hypoxia and improve the efficacy of EGFRVii-targeted CAR T cells. Created with BioRender.com

The importance of boosting the immune response and limiting the immunosuppression condition is undoubtedly one of the challenges for GBM treatment. This condition includes a detailed knowledge of the temporal and spatial interactions among immune cells, the endothelium, the GBM, and the anatomical and functional properties of the brain barriers (Fig. 3).

For this reason, recent research has focused on the in-silico multidimensional models integrating spatially resolved and single-cell gene expression analysis to provide a detailed spatial resolution of the distribution of immune system cells in tumor tissue. With this technique, Ravi and colleagues described the role of specific cellular subsets that release soluble factors (such as interleukin-10), drive T-cell exhaustion, and contribute to the generation of an immunosuppressive tumor microenvironment [93]. One of the mechanisms of crosstalk between tumor and immune infiltrating cells which models the tumor microenvironment, has been elegantly described by Takenaka and colleagues, showing that kynurenine secreted by GBM cells recruit TAMs through the aryl hydrocarbon receptor-CCR2-CCL2 axis, thus driving the expression of the ectonucleotidase CD39 and the consequent adenosine production in TAMs, therefore promoting CD8 T cell dysfunction [95]. In addition to CD39, the role of another immune-metabolic checkpoint, CD73 has been recently explored, describing that CD73 inhibition can be a promising strategy to potentiate GBM immunotherapy [96].

An additional mechanism contributing to impaired CD8 T cell activation in GBM involves a distinct subset of regulatory CD103 + T cells. CD103 (integrin αE) is typically associated with tissue-resident T cells. In the GBM microenvironment, regulatory CD103⁺ T cells are highly abundant and exert potent immunosuppressive activity [97]. Notably, upon combined anti–PD-1 therapy and radiotherapy, these CD103⁺ regulatory T cells have been shown to limit cytotoxic CD8⁺ T cell responses, thereby constraining the efficacy of immune checkpoint blockade [98].

To make a detailed analysis of the T-cell subset colonizing the GBM, Mathevson and colleagues used flow cytometry to describe that a large fraction (> 90%) of GBM-infiltrating CD8 T cells and most CD4 T cells express the NK inhibitory receptor CD161, whereas only a small subset of CD161^+^ cells can be detected in the peripheral blood of the same patient [99]. The importance of this discovery relies on the CD161 role as an inhibitory receptor for tumor-specific T cells and could be a potential target for immunotherapy.

A potential strategy to enhance CD8⁺ T cell infiltration into the tumor mass relies on the disruption of endothelial phosphoglycerate dehydrogenase (PHGDH), an enzyme that plays a critical role in the de novo serine biosynthesis pathway and fuels the formation of aberrant vascular structure and hypoxic microenvironment (Fig. 3) [100].

Recent advances in spatial transcriptomics applied to surgical resections of GBM have revealed that CD8⁺ Granzyme (GZM) K⁺ effector T cells represent a considerable fraction of tumor-infiltrating CD8⁺ lymphocytes. Interestingly, regions enriched in GZMK^hi^ T cells appear to be spatially restricted to extravascular compartments. These niches are marked by the presence of vascular leptomeningeal cells and endothelial cells, highlighting the importance of specific microenvironmental contexts in supporting the accumulation and possibly the functional specialization of these effector T cells within GBM [101].

Given the role of CD8⁺ T cells in GBM progression, understanding the mechanisms underlying their trafficking across the blood–tumor barrier (BTB) and their interactions with GBM cells is critical for developing strategies to overcome immunosuppression and enhance their cytotoxic activity. (Fig. 3).

## Experimental in vitro and in vivo approaches to study the BTB

### In vitro models

Deciphering the complex crosstalk between GBM and the brain microvasculature requires experimental platforms capable of capturing their dynamic and multifaceted interactions, making in vitro models an indispensable tool. Owing to their controllable environment and the use of different cell types, the effects of cell-cell interactions, the role of secreted factors, or the reaction to chemical compounds can be investigated in more detail in in vitro experiments, often beyond what is achievable in vivo.

In addition, these systems allow the use of human-derived cells, whereas in vivo approaches are largely limited to xenograft models in immunocompromised animals. Importantly, given the limited accessibility of this specific anatomical site and the highly specialized microenvironment that forms at its interface, in vitro disease modelling represents a crucial tool for dissecting underlying mechanisms and advancing translational research.

#### Human Induced pluripotent stem cells (iPSCs) and 3D platformsHuman Induced pluripotent stem cells (iPSCs) and 3D platforms

In the complex landscape of in vitro models aimed at recapitulating the structural and functional complexity of the brain vasculature, stem cell–derived systems are increasingly gaining prominence. Recently, hiPSCs have emerged as a versatile platform, capable of generating endothelial cells [102], pericytes [103], and astrocytes [104] that closely mimic the architecture and functionality of the human BBB. Furthermore, human GBM cells derived from iPSCs [105] have recently revolutionized the range of cellular resources available for in vitro research and enable patient-specific studies for personalized medicine [106].

hiPSCs and GBM cells have proven highly useful for generating tumoroids or assembloids that recapitulate key molecular and phenotypic features of GBM [107]. Through coculture strategies and exposure to tumor-conditioned cues, these models highlight the instructive role of the TME in shaping stemness and cellular behavior [106, 108], while microfabricated 3D platforms further enable the controlled integration of multiple cell types, including immune components [109, 110], allowing functional studies of adhesion, invasion, and chemotaxis (e.g., toward SDF1α [111]). Recently, these models have been employed to evaluate the efficacy of CAR-based immunotherapies. In particular, targeting pericytes has emerged as a potential strategy, with a CD19 CAR-engineered iNK cell approach proposed in a GBM spheroid model to deplete pericytes [72]. This approach uses an “off-the-shelf” NK cell platform originally developed for CD19⁺ B-cell malignancies, offering potential advantages over CAR-T cells, including reduced toxicity and faster production.

Despite major advances in 3D modeling of the tumor microenvironment, the lack of functional vascularization and perfusion limits the faithful recapitulation of the tumor microenvironment, restricting these models primarily to the study of GBM–brain cell interactions.

#### Microfluidic platforms

Microfluidic platforms have increasingly demonstrated superior physiological relevance compared with conventional static systems. By integrating continuous fluid flow, physiologically relevant shear stress, and compartmentalized microchannels that more closely mimic vascular architecture, these “BBB-on-chip” models recapitulate key aspects of in vivo hemodynamics [112, 113]. Under dynamic conditions, endothelial cells display improved tight junction organization, higher barrier integrity as measured by transendothelial electrical resistance (TEER), and permeability profiles that more closely resemble in vivo behavior [114, 115]. Dynamic perfusion is critical not only to maintain endothelial phenotype and barrier function but also to provide sustained nutrient delivery and waste removal, which are necessary to support the viability and metabolic activity of heterogeneous cell populations over extended culture periods [116].

**Fig. 4:**
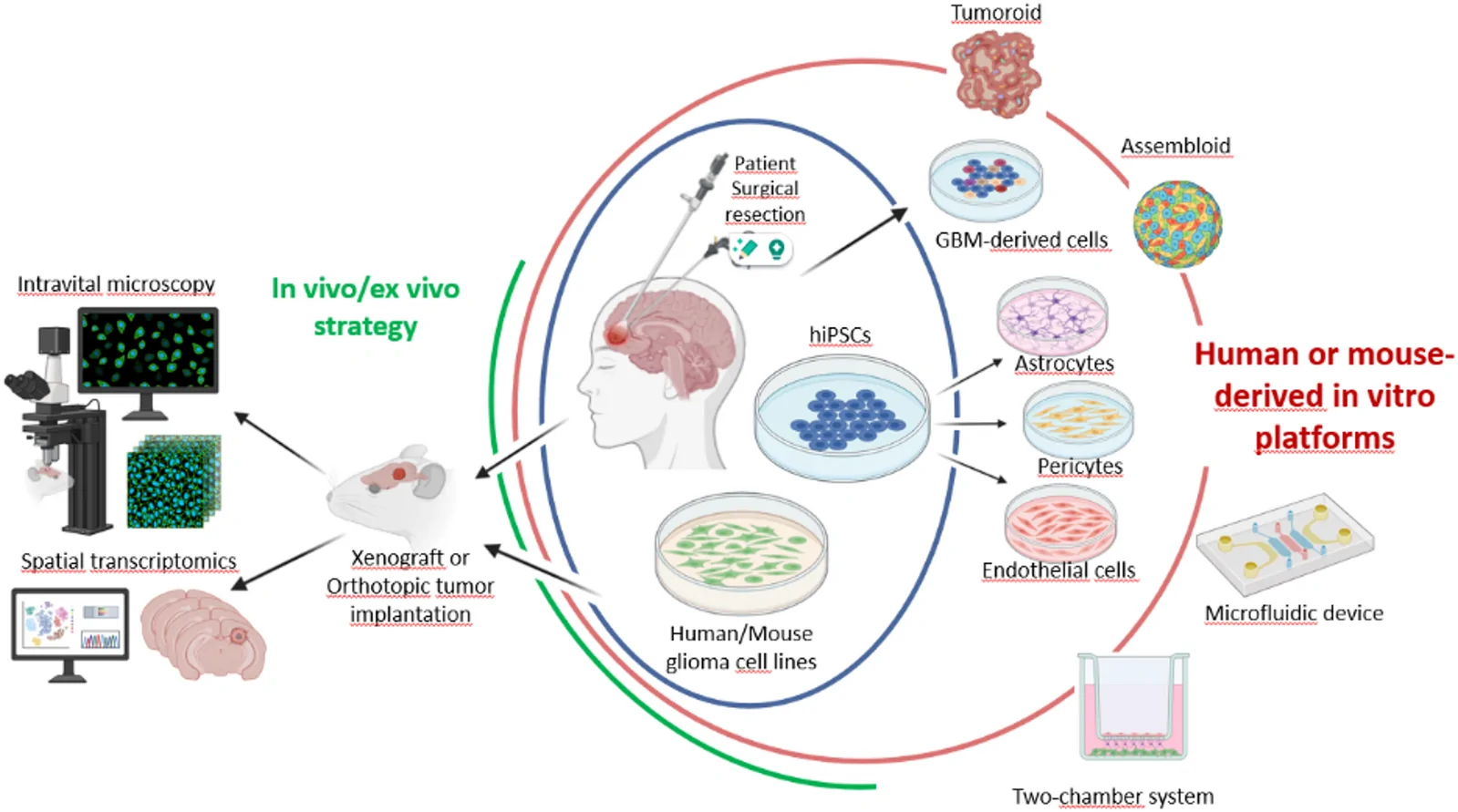
In vitro and in vivo platforms for GBM studies. Patient-derived surgical specimens, hiPSCs, and human or murine glioma cell lines (circled in blue) represent the principal sources for generating 2D and 3D in vitro platforms (circled in red), enabling integrated investigation of brain tumor cells and NVU components. hiPSC-derived NVU models provide physiologically relevant, patient-specific systems that, in combination with GBM cells, support mechanistic studies and the development of precision therapeutic strategies. Primary patient cells and established cell lines (indicated by the green line) are also used to generate xenograft and orthotopic mouse models, including murine models suitable for intravital microscopy and spatial transcriptomics studies. Created with BioRender.com

Importantly, these microfluidic BBB models have been shown to recapitulate key aspects of human/mouse neurovascular physiology, allowing the interactions with astrocytes and pericytes that are essential for accurate modeling of barrier function and dysfunction [117, 118]. Furthermore, these platforms, combining cellular and acellular elements with nanoporous membranes and iPSC-derived patient-specific cells, provide a physiologically relevant and personalized framework to investigate crosstalk within the BTB (Fig. 4).

### In vivo Models and Two-Photon Intravital microscopy

The following section presents the currently most used experimental strategies suitable for investigating the interactions between GBM cells and the BBB.

The in vivo studies predominantly rely on murine models, with syngeneic mouse models being the most widely used. In these models, murine glioma cells are implanted into genetically matched, immunocompetent mice [119]. Although tumor implantation surgery inevitably perturbs brain structures, syngeneic models are invaluable for investigating tumor progression, immune cell infiltration, and tumor–immune interactions within a functional immune system.

However, investigating the specific GSCs contribution remains challenging in these models, as murine glioma cells generally exhibit fewer stem-like features and reduced invasive/infiltrative behavior compared with human GSCs. As a result, important aspects of GBM heterogeneity and glioma stem cell biology may not be fully recapitulated. In this regard, spontaneous transgenic glioma models may provide a more faithful representation of gliomagenesis, tumor heterogeneity, and disease progression, although their broader application is limited by the specialized expertise, breeding strategies, and resources required for their generation and maintenance.

In this context, two-photon intravital microscopy (2P‑IVM) is an indispensable tool in this context, providing high-resolution, real-time imaging of neurovascular architecture and cellular dynamics in vivo (Fig. 4). Endothelial markers such as VE‑cadherin serve to delineate vascular junctions and define fiduciary landmarks [120], while aquaporin‑4 (AQP4) labels perivascular astrocytic endfeet, enabling precise mapping of glial–vascular interactions [121]. The combined use of these markers would allow for detailed visualization of BTB heterogeneity, revealing regions of leaky and intact vasculature and shedding light on the BTB microenvironment.

Human patient-derived xenograft (PDX) models provide a complementary approach. In these models, tumor samples obtained from surgical resections of GBM patients are implanted into immunodeficient mice, allowing the preservation of patient-specific tumor characteristics and molecular heterogeneity. PDX models are particularly valuable for studying intrinsic tumor biology, but the absence of a totally functional immune system limits their use in investigating the complex interactions that occur at the BTB level.

Spatial transcriptomics has rapidly emerged as a fundamental tool for dissecting the spatial architecture and cellular heterogeneity of GBM, particularly in anatomically and functionally complex interfaces such as the BTB. Unlike traditional bulk or single‑cell sequencing, spatial transcriptomics preserves gene expression within intact tissue architecture, enabling researchers to map transcriptional programs to specific histological features and microenvironments. In GBM, spatial transcriptomic studies have revealed pronounced segregation of tumor cell states and distinct expression niches associated with perivascular and invasive regions, highlighting the spatial organization of cells within the tumor microenvironment [122–124].

In the context of the BTB, spatial transcriptomics enables the mapping of tumor cells, stromal components, and vascular structures at high resolution, revealing their interactions across distinct microanatomical zones and informing on tumor distribution, vascular remodeling, and immune infiltration. This spatially resolved insight is crucial for understanding how heterogeneous cellular niches drive GBM progression and influence therapeutic response.

## Conclusions

In this review, we summarized current knowledge on the interactions between the brain microvasculature and the GBM microenvironment, highlighting how this highly specialized anatomical interface is profoundly remodeled during disease progression.

Currently, the standard of care for GBM consists of maximal safe surgical resection followed by radiotherapy and temozolomide chemotherapy. Although substantial efforts are being directed toward the development of novel therapeutic strategies, our current understanding of treatments specifically targeting the cellular and molecular interactions within the BTB remains too limited to support a comprehensive discussion. Therefore, this review primarily focuses on the biological mechanisms underlying GBM–BTB interactions rather than on therapeutic approaches.

In GBM, the BBB evolves into a distinct structure referred to as the BTB, shaped by tumor cell colonization of the neurovascular unit and characterized by extensive cellular and extracellular matrix remodeling. A central focus was placed on the perivascular niche as a critical hub sustaining GSCs, whose interactions with endothelial cells, pericytes, and the extracellular matrix promote stemness, therapy resistance, and tumor recurrence. We discussed how intercellular communication mechanisms, including tumor microtubes (TMs), further enhance tumor adaptability and resistance to chemotherapy.

Although immune cell infiltration is observed in surgical resections from GBM patients as well as in syngeneic mouse models, persistent BTB properties restrict drug delivery and compromise therapeutic efficacy. This represents a key consideration for the development of more effective immunotherapeutic strategies in GBM.

Collectively, these findings underscore the need for advanced disease modeling approaches that accurately recapitulate neurovascular physiology and tumor–barrier interactions. The use of murine models expressing specific endothelial and astrocytic markers in syngeneic systems would provide a comprehensive assessment of tumor progression, allowing better characterization of cellular interactions involved in BTB formation. It would also enable high-resolution mapping of BTB heterogeneity, identifying areas of both disrupted and preserved vascular integrity, thereby providing further insight into the BTB microenvironment.

## Data Availability

No datasets were generated or analysed during the current study.

## Abbreviations

APCs: Antigen–Presenting Cells
BBB: Blood–Brain Barrier
BAMs: Border–Associated Macrophages
BTB: Blood Tumor Barrier
CNS: Central Nervous System
EC: Endothelial Cell
ECM: Extracellular Matrix
GBM: Glioblastoma
GZM: Granzyme
GSCs: Glioma Stem Cells
hiPSCs: human–Induced Pluripotent Stem Cells
HMECs: Human Microvascular Endothelial Cells
hCMECs: human Cerebral Microvascular Endothelial Cells
NSC: Neural Stem Cells
PDX: Patient–Derived Xenograft
PHGDH: Phosphoglycerate Dehydrogenase
POSTN: Periostin
PVN: Perivascular Niche
SDF1α: Stromal–Derived Factor 1α
SMCs: Smooth Muscle Cells
SVZ: Subventricular Zone
TGFβRIII: TGF–βreceptor III
TMs: Tumor Microtubes
TNT: Tunneling Nanotubes
VEGF: Vascular Endothelial Growth Factor
